# A Ca^2+^-Mediated Switch of Epiplakin from a Diffuse to Keratin-Bound State Affects Keratin Dynamics

**DOI:** 10.3390/cells11193077

**Published:** 2022-09-30

**Authors:** Sonia Ratajczyk, Corinne Drexler, Reinhard Windoffer, Rudolf E. Leube, Peter Fuchs

**Affiliations:** 1 Max Perutz Labs, Department of Biochemistry and Cell Biology, University of Vienna, Vienna Biocenter Campus (VBC), A-1030 Vienna, Austria; 2 Vienna Biocenter PhD Program, A Doctoral School of the University of Vienna and Medical University of Vienna, A-1030 Vienna, Austria; 3 Institute of Molecular and Cellular Anatomy, RWTH Aachen University, Wendlingweg 2, 52074 Aachen, Germany

**Keywords:** cytoskeleton, keratin, intermediate filaments, plakin protein family, epiplakin

## Abstract

Keratins exert important structural but also cytoprotective functions. They have to be adaptable to support cellular homeostasis. Epiplakin (EPPK1) has been shown to decorate keratin filaments in epithelial cells and to play a protective role under stress, but the mechanism is still unclear. Using live-cell imaging of epithelial cells expressing fluorescently tagged EPPK1 and keratin, we report here an unexpected dynamic behavior of EPPK1 upon stress. EPPK1 was diffusely distributed throughout the cytoplasm and not associated with keratin filaments in living cells under standard culture conditions. However, ER-, oxidative and UV-stress, as well as cell fixation, induced a rapid association of EPPK1 with keratin filaments. This re-localization of EPPK1 was reversible and dependent on the elevation of cytoplasmic Ca^2+^ levels. Moreover, keratin filament association of EPPK1 led to significantly reduced keratin dynamics. Thus, we propose that EPPK1 stabilizes the keratin network in stress conditions, which involve increased cytoplasmic Ca^2+^.

## 1. Introduction

Keratin filaments represent the main class of cytoplasmic intermediate filaments in epithelial cells. They form a large protein family whose members are encoded by more than 50 genes [1]. Together with actin filaments and microtubules, epithelial keratins form a cytoskeletal network that maintains internal cellular organization and tissue integrity. However, keratins not only perform essential structural functions, ensuring the stabilization of the cell and its internal components, but also play a cytoprotective role, exemplified by many disorders caused by keratin gene mutations [1,2]. Diseases associated with keratin mutations may lead to severe changes in the architecture of the keratin network through alterations in their expression levels, intracellular localization, and post-translational modifications (PTMs). There is growing evidence that these changes can influence many crucial processes, such as tumor growth and metastasis, cell migration and polarization, and the development of infections [2]. The fact that keratin mutations disturb dynamic processes such as cell migration implies that, besides providing rigidity and stability to the cell, keratins have to remain flexible and prone to rapid reorganization to allow adaptation to changing microenvironments and restructuring processes [3].

The molecular basis of this flexibility was studied by detailed investigations of the assembly and disassembly of keratin filaments, finally leading to the proposal of the keratin cycle [4]. This multistep process is characterized by spatially defined assembly and disassembly of keratin filaments and may be controlled by PTMs of keratins [5]. In addition, it has been suggested that keratin-binding proteins such as plakins are involved in these processes [6,7].

One candidate for such a protein is epiplakin (EPPK1), an extraordinary member of the plakin family. Plakins typically consist of distinct structural domains, notably actin- and microtubule-binding domains [8]. Further characteristics are the presence of a plakin domain and of a plakin repeat domain (PRD) [9]. The latter, which is missing in periplakin, facilitates the interaction with intermediate filaments [10,11]. EPPK1 is unusual since it lacks actin- and microtubule domains and a plakin domain and consists solely of PRDs [8,12]. Detailed analysis of the human and murine EPPK1 genomic locus revealed a high degree of polymorphism due to the presence of several EPPK1 variants comprising 9 to 17 PRDs as a consequence of variable copy numbers of almost identical PRDs at the C-terminus [13,14]. The expression of EPPK1 is restricted to epithelial tissues with the highest expression levels in the epidermis [12,15]. Co-localization of EPPK1 with keratin filaments was demonstrated for different cultured cell lines and primary cells [16,17]. Using blot overlay assays, several PRDs of mouse EPPK1 were shown to bind to the simple epithelial keratins 8 (K8) and K18 and the epidermal K5 and K14 [17,18], but also to other intermediate filament polypeptides such as vimentin [16,19]. EPPK1-deficient (EPPK^−/−^) mice showed no obvious phenotype [20,21]. However, a phenotype was observed when liver and pancreas stress models were applied to EPPK^−/−^ mice. Induced pancreatitis and liver disease, which led to parallel upregulation of keratin and EPPK1 in control mice, resulted in an aggravated course of disease and the formation of keratin aggregates in EPPK^−/−^ mice [18,22]. These phenotypes suggest that EPPK1 has a protective keratin organizing function. Until now, however, it is not understood how EPPK1 fulfills this role in stressed cells. In the present study, we used time-lapse microscopy to investigate the interaction of EPPK1 and keratins in stressed and homeostatic cells. We present the completely unexpected finding that EPPK1 proteins do not co-localize with keratin filaments in homeostatic conditions as reported before but are diffusely distributed throughout the entire cell. Yet, EPPK1 is rapidly and reversibly recruited to keratin filaments in conditions leading to elevated cytoplasmic Ca^2+^ levels such as stress. In addition, we show that the re-localization of EPPK1 to keratins slows down keratin dynamics.

## 2. Materials and Methods

### 2.1. Cell Culture

Cells were grown in DMEM (high glucose and L-Glutamine, Gibco, Paisley, United Kingdom) supplemented with 10% FCS and Penicillin-Streptomycin and incubated at 37 °C with 5% CO_2_. Cells were split three times a week and were kept in sub-confluent conditions. All cell lines used and generated are listed in Table 1.

### 2.2. Immunofluorescence Microscopy

Cells were washed with PBS and fixed for 20 min using 4% PFA in PBS. Next, cells were incubated with 50 mM NH_4_Cl, permeabilized with 0.5% Triton-X-100, washed with PBS, and stained with EPPK1 (EPPK1#24)- specific antibodies (see Table 2). Samples were imaged with an inverse confocal microscope (Zeiss LSM 980, Zeiss, Jena, Germany) with a Plan-Apochromat 63×/1.4 Oil DIC objective using Zeiss ZEN 3.3 acquisition software.

### 2.3. Co-Sedimentation Assay

Cells were grown to full confluence on 6 cm cell culture dishes. Subsequently, cells were washed once with DPBS (Ca^2+^ and Mg^2+^-free PBS) and 1.5 mL of cell lysis buffer (PBS with CaCl_2_; 0.1% Triton-X-100) with or without 5 mM EGTA was added to the cells. Cells were scraped off the dish, transferred into a 1.5 mL tube, and incubated at room temperature (RT) for 5 min. Cell lysates were centrifuged at 20,238× *g* at 4 °C for 12 min. Total supernatants (1.5 mL) representing the soluble protein fractions were aliquoted and stored at −80 °C or immediately used for further analyses. Pellets containing the insoluble protein fraction were solubilized in 100 µL of 8 M urea by shaking on a thermomixer with 1000× rpm at RT. Samples were sonicated (100% power, 5 cycles, 40 s) to destroy genomic DNA and fully dissolve the pellet. Then, 50 µL loading dye was added, and pellet samples were aliquoted and stored at −80 °C or immediately used for further analyses. Typically, 40 µL of the supernatant and 4 µL of the pellet samples were loaded for SDS-PAGE analysis to enable comparisons of protein fractions.

### 2.4. Preparation of Protein Lysates from Cultured Cells

Cells used to obtain protein lysates were grown on 10 cm dishes until full confluence. Cells were washed once with DPBS (Ca^2+^ and Mg^2+^-free PBS), 1.5 mL of cell lysis buffer was added (50 mM HEPES; 100 mM NaCl; 5 mM MgCl_2_; 1 mM EGTA; 2.5% Triton X 100; 0.1 mM DTT; 1 mM PMSF; 0.5 mg/mL DNaseI; 0.2 mg/mL RNase; Protease Inhibitor Cocktail: cOmplete™ ULTRA Mini EDTA-free Tablets, Roche, Mannheim, Germany), and scraped off the dish, transferred to a 1.5 mL tube, and incubated at RT for 5 min. Subsequently, lysates were pushed through a 27G needle 10 times to fragment the remaining genomic DNA. Protein lysates were aliquoted and stored at −80 °C or were immediately used for downstream analysis by SDS PAGE.

### 2.5. SDS-PAGE

Protein samples were mixed with 5× SDS loading dye (Tris-HCl, pH 6.8 390 mM, DTT 485 mM, SDS 10%, bromophenol-blue 0.1%, glycerol 50%) and incubated at 95 °C for 5 min or at 60 °C for 10 min if the proteins had been re-solubilized with 8 M urea, and were loaded on SDS-polyacrylamide gels (NuPAGE™ 3 to 8%, Tris-Acetate gels (Invitrogen, Carlsbad, CA, USA) for EPPK1 and 12% polyacrylamide gels for keratins. Gel electrophoresis was performed with 20 mA for 1.5 h for keratin gels and with 150 V for 5 h for EPPK1 gels (SDS running buffer for 12% gels: 25 mM Tris, 250 mM glycine, 0.1% SDS; running buffer for NuPAGE™ 3 to 8%, Tris-Acetate gels: NuPAGE™ Tris-Acetate SDS Running Buffer, Invitrogen, Carlsbad, CA, USA).

### 2.6. Western Blot

After gel electrophoresis, separated proteins were blotted onto a nitrocellulose membrane using a wet blot transfer overnight at 4 °C with 25 V (Transfer buffer: 25 mM Tris, 192 mM Glycine). Membranes were stained with Ponceau Red to verify successful protein transfer. Membranes were blocked with 5% BSA in PBS-T for 1 h and subsequently incubated with primary antibody solution (RFP antibody 1:2000 for short-tagged EPPK1; Troma 1 antibody 1:1000 for K8; human EPPK1 antibody (EPPK#66) 1:2000 for endogenous EPPK1) for 3 h (see Table 2). Incubation with secondary antibodies (see Table 3) was performed for 2 h (anti-mouse HRPO 1:20,000 for RFP antibody; anti-rat HRPO 1:20,000 for Troma 1 antibody; anti-rabbit HRPO 1:20,000 for EPPK1 antibody). After incubation of the membrane with Clarity ECL Western Blotting substrate (BioRad, Hercules, CA, USA), chemiluminescence signals were analyzed using a ChemiDoc device (BioRad, Hercules, CA, USA). 

### 2.7. Cloning of cDNAs Encoding Short EPPK1

A vector encoding pmScarlet C1 was purchased from Addgene (item ID 111543). Primers for amplification of EPPK1 PRDs 1–8 (5′-ATGAGTGGCCACACCTTGCCTC-3′, 5′-CTCCTCGCTCAGCTGGTGCAC-3′) and PRD 13 (5′-GTGCACCAGCTGAGCGAGGAG-3′, 5′-CTGTAGAGAGAGAGAAAGAAATAG-3′) were designed based on the EPPK1 coding reference sequence (NCBI Ref. Sequence: NG_033825.1) and according to [13]

The cDNA encoding short human EPPK1 was generated using NEBuilder^®^ HiFi DNA Assembly Master Mix (New England Biolabs, Ipswich, MA, USA, cat. no. E2621S). The EPPK1 regions encoding PRDs 1–8 and PRD 13 were amplified with PCR, adding overlaps according to the manufacturer’s guidelines and assembled into the mScarlet C1 vector.

Plasmids harboring cDNAs coding for mScarlet-short EPPK1 were sequenced and linearized by digestion with the restriction endonuclease *Pci*I prior to transfections of cells performed to generate stable cell lines. 

### 2.8. Generation of Stable Cell Lines

For generating stable cell lines expressing mScarlet-short EPPK1, 1 million AK 13-1 WT, EpiKO/AK, or A-431 cells were electroporated with a Neon Transfection System (Invitrogen, Carlsbad, CA, USA) according to the instructions of the manufacturer (electroporation conditions: 1100 V, 20 ms, 2 pulses) using 5–10 µg of linearized plasmid DNA coding for mScarlet-short EPPK1. One week after transfection, mScarlet positive cells were sorted by FACS (BD FACSAria™ IIIu). Cell clones were generated by expanding sorted single cells. Expression of the transgene was confirmed by microscopy and Western blot analysis.

For generation of a cell line expressing a Ca^2+^ sensor, 1 million A-431 cells expressing mScarlet short EPPK1 were electroporated with a Neon Transfection System using 10 µg of linearized pN1-GCaMP6m-XC vector (Addgene item ID 111543). One week after transfection, 500 µM CaCl_2_ was added to the cell suspension after trypsinization but before sorting to increase the fluorescence signal level of the Ca^2+^ sensor and thereby improve the efficiency of cell sorting. Cellular membrane breaches caused by trypsinization enabled Ca^2+^ influx into the cells and thereby increased the fluorescence signal in the cells, which had stably incorporated the sensor. After stimulation with 500 µM CaCl_2_ for 15 min, GCaMP6m (green) and mScarlet-positive cells were sorted with FACS (BD FACSAria™ IIIu) and expanded in culture. Transgene expression was confirmed by microscopy.

### 2.9. Generation of CRISPR/Cas9 Knock-in and EPPK1^−/−^ Cell Lines

Selection and testing of gRNAs were performed by the genome engineering services of the VBCF Protein Technologies Facility at the Vienna Biocenter (Vienna, Austria).

### 2.10. EPPK1^−/−^

EPPK1^−/−^ cell lines were generated using CRISPR/Cas9 Non-Homologous End Joining [23]. One million AK 13-1 WT cells were electroporated with the Neon Transfection System according to manufacturer instructions (electroporation conditions: 1100 V, 20 ms, 2 pulses) with 12 µg of gRNA (5′-GGCTATCCTGACCCCTACGG-3’) and 5 μg of Cas9 protein. Cell clones were generated by single-cell dilution and the successful generation of Indels was evaluated by sequence analysis using the online genome editing analysis tool “TIDE-Web tool”.

### 2.11. Knock-in

The EpiKI/AK cell line was generated using a CRISPR/Cas9 homology-directed repair strategy [24]. An EPPK1 C-terminal repair construct was generated by a series of PCR reactions carried out using Q5 polymerase (New England Biolabs, Ipswich, MA, USA). The DNA sequence, consisting of a left homology arm, linker, mScarlet, linker, 6×HIS-tag, and right homology arm, was cloned into the pUC19 vector using NEBuilder^®^ HiFi DNA Assembly (New England Biolabs, Ipswich, MA, USA). One million AK 13-1 WT cells were electroporated with 12 µg guide RNA (5′-CCGTGCAGTTTTCTGCAACT-3′), 5 µg of Cas9 protein and 1 µg repair construct with the Neon Transfection System according to manufacturer instructions (electroporation conditions: 1100 V, 20 ms, 2 pulses). Two days after electroporation, mScarlet positive cells were sorted with FACS (BD FACSAria™ IIIu) either collectively or as single clones and expanded in culture.

### 2.12. Live Cell Imaging

For live-cell imaging, 30,000 cells were seeded on laminin-rich coated 3.5 cm glass-bottom dishes (WPI, Sarasota, FL, USA, cat. no. FD35-100). One hour after seeding, the medium was exchanged to FCS-free DMEM (high glucose and L-Glutamine, Gibco, Paisley, United Kingdom) supplemented with Penicillin-Streptomycin. The cells were incubated for an additional 23 h. Prior to imaging, the medium was exchanged for DMEM medium without phenol red and FCS (FluoroBrite DMEM, Gibco, Paisley, United Kingdom). Live-cell imaging was performed using a spinning disc microscope (Zeiss Axio Observer Z1, inverse, fully motorized, with hardware autofocus, Yokogawa CSU-X1-A1 Nipkow spinning disc unit) and a Plan-Apochromat 63×/1.4 DIC Oil objective. For UV irradiation experiments, an EC Plan-Neofluar 100×/1.30 Oil Iris objective was used. The microscope is equipped with full environmental control, and imaging took place at 37 °C. EGFP-tagged HK13 and mScarlet-tagged short EPPK1 were illuminated with a 488 nm laser (10% intensity, exposure time 150 ms) and a 561 nm laser (25% intensity, exposure time 200 ms), respectively. The signal was detected with a sCMOS: 2× pco.edge 4.2 camera system (2048 × 2048 pixel, 6.45 µm pixel size, 16 bit, 100 fps, QE 70%). Images were acquired with VisiView 5.0.0.11 acquisition software (Visitron Systems, Puchheim, Germany).

### 2.13. Time-Lapse Microscopy of Cells during Fixation

EpiKI/AK and shEpi/AK cells were cultivated as described above. One day after seeding, cells were imaged before and after a washing step with PBS, and after the addition of 0.5%, 2%, and 4% PFA/PBS solution. For methanol fixation, cells were imaged before and after treatment with ice-cold methanol.

### 2.14. Time-Lapse Microscopy of Cells during Drug Treatment

Cells were treated with various drugs during live-cell imaging (for details see Table 4). Untreated cells in 2 mL of FluoroBrite medium were monitored for 2–3 time points. Subsequently, 1 mL of the medium was removed from the dish and 1 mL of medium containing 2× concentrated drug was added. Cells were imaged in multiple z-planes (slice interval 0.75–0.8 µm) with various time intervals from 30 s to 2 min for 10–30 min. Plots showing the intensities of EPPK1 and keratin fluorescent signals before and after treatment were generated using ImageJ software.

### 2.15. UV-A Irradiation

For local UV-A treatment, cells were irradiated with UV-A during live-cell imaging with a Spinning Disc microscope. Cell culture conditions and imaging parameters for monitoring fluorescently-tagged keratin and short EPPK1 are described above. For UV-A irradiation, a 355 nm laser (355 nm passively Q-switched pulsed ablation laser, 16 mW average power) was used. An area of 50 × 50 pixels was irradiated with 40% laser intensity and 10 ms irradiation time per pixel to induce EPPK1 association with keratins. The applied UV-A irradiation corresponds to a UV dosage of 16 J/cm^2^, which lies within physiological UV-A irradiation dosages (up to 60 J/cm^2^) [25]. Images were taken every 90 s, and a z-stack was acquired for every time point. UV-A irradiation was applied once after three images were acquired.

For whole-cell UV-A irradiation, the entire cell culture dish was illuminated for 10 min with a total dosage of 40 J/cm^2^, emitted by a Waldmann F15 T8 tube, which is equipped with a UVA-1 light source (340–390 nm). Cells were monitored by confocal live microscopy using full environmental control and imaged before and after UV-A irradiation.

### 2.16. Monitoring of the Keratin Flow

The cell culture conditions and imaging parameters used are described above. Images were taken every 90 s, and z-stacks (14 z-slices) were acquired for every time point. Keratin dynamics was monitored for 15 time points under physiological conditions (non-keratin-associated EPPK1). Subsequently, cells were treated with 50 nM Tg to induce EPPK1 association with keratin filaments and keratin dynamics was monitored for another 15 time points.

For the analysis of each recorded stack, a maximum projection was generated. For each experiment, the keratin flow of time points 1–10 (before Tg addition) was compared with the keratin flow of time points 21–30 (after Tg addition). The resulting videos were registered to remove potentially existing XY shift using the stackreg plugin (http://bigwww.epfl.ch/thevenaz/stackreg/, (accessed on 26 January 2022)). The CMove program was used to calculate keratin flow as described in [26]. The analyzed cells were selected for further analysis based on the calculation of shrinkage. The cell size in each movie’s first and last image was compared. Only cells with less than 15% shrinkage were used for the statistical evaluation of keratin flow before and after treatment with Tg. Data represent at least three independent experiments. Statistical evaluation was performed using the Wilcoxon matched-pairs signed-rank test in GraphPad Prism software; ns *p* > 0.05, **** *p* ≤ 0.0001.

### 2.17. Laminin-Rich Coating

For live cell imaging, cells were cultivated on a laminin-332-containing matrix derived from 804G cells. 804G cells were detached with sterile 20 mM NH_4_OH one day after they reached confluency. Cell debris was removed and the dishes were washed three times with 4 mL sterile ddH_2_O to remove the remaining cell debris and once with 4 mL PBS without Ca^2+^ and Mg^2+^. The dishes were immediately used or stored filled with PBS supplemented with 10% DMSO at −20 °C.

### 2.18. Intensity Correlation Analysis

shEpi/AK cells were incubated in FluoroBrite DMEM with or without 20 µM BAPTA AM. After 40 min of incubation, cells were treated with 200 nM of Tg. Images of mScarlet-short EPPK1 and HK13-EGFP before and after Tg treatment were acquired with a spinning disc microscope at 2 min intervals. The acquired z-stack images were converted to maximum intensity projections. The regions of interest for analysis were chosen by thresholding a keratin signal with the Huang thresholding method in ImageJ [27]. The intensity correlation quotients for individual time frames in the keratin and short EPPK1 channels were determined using the Intensity Correlation Analysis ImageJ plugin [28]. Data represent at least three independent experiments. Statistical evaluation was performed using the student *t*-test in GraphPad Prism software; ns *p* > 0.05, **** *p* ≤ 0.0001.

## 3. Results

### 3.1. EPPK1 Is Diffusely Distributed throughout the Cytoplasm of Vital Cells under Standard Cell Culture Conditions but Rapidly Re-Localizes to Keratin Filaments during Fixation

**Figure 1:**
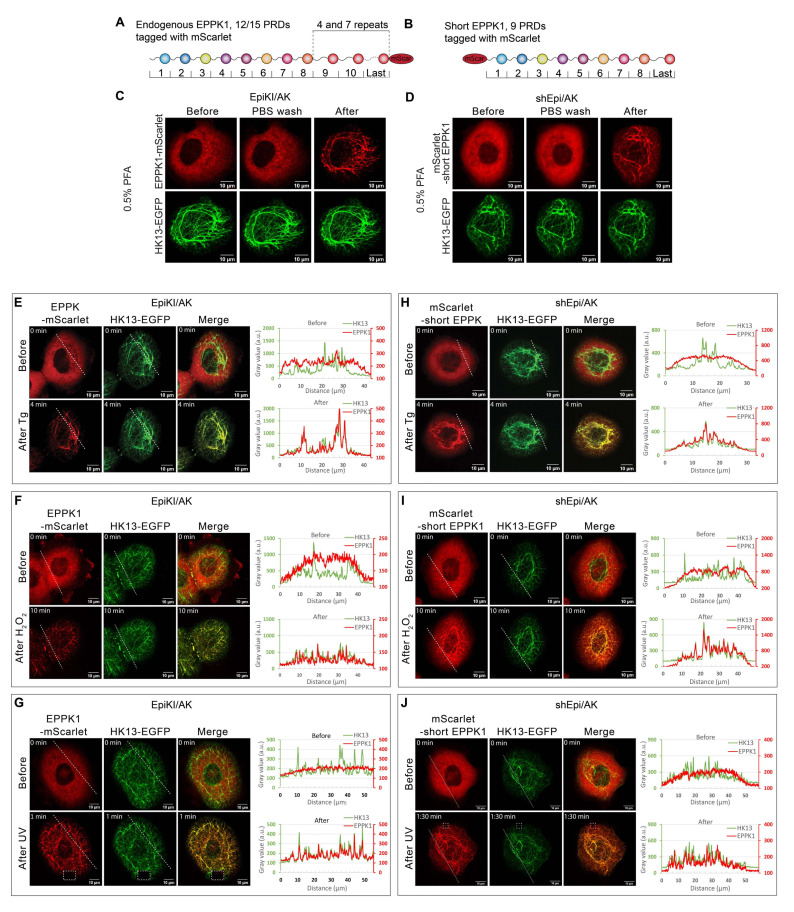
EPPK1 is diffusely distributed in the cytoplasm of living A-431-derived cells and rapidly associates with keratin filaments during fixation with PFA and after stress induction. (**A**,**B**) Schematic representation of fluorescently tagged EPPK1 variants used for live-cell imaging in AK 13-1 cells. (**A**) Both endogenous *EPPK1* alleles of AK 13-1 cells were tagged with an mScarlet-encoding gene fragment using CRISPR/Cas9 HDR at their carboxytermini. The corresponding fusion proteins differ in size due to copy number variations in their almost identical C-terminal PRDs (4 versus 7), encompassing either 12 or 15 PRDs. (**B**) An EPPK1 variant (short EPPK1) consisting of the first eight N-terminal PRDs and the last of the identical C-terminal PRDs was N-terminally tagged with mScarlet. (**C**,**D**) Live-cell imaging during PFA fixation of EPPK1-mScarlet (red) and HK13-EGFP (green) in EpiKI/AK cells (**C**) or shEpi/AK cells (**D**). The respective left panels show cells before fixation, the middle panels show cells after removal of medium and subsequent PBS wash, and the right panels show cells after 0.5% PFA addition. EpiKI/AK cells (**E**–**G**) and shEpi/AK cells (**H**–**J**) were treated with 200 nM Tg (**E**,**H**), 2.5 mM H_2_O_2_ (**F**,**I**)_,_ and 16 J/cm^2^ UV-A light pulse (**G**,**J**). In standard cell culture conditions (**upper panels E**–**J**) EPPK1 localization was diffuse and not keratin-associated. Plots on the right side of the panels display fluorescent signal intensities (arbitrary units) of EPPK1 (red) and keratin (green) along the dashed lines (from left to right) indicated in the image panels. The mainly non-overlapping keratin and EPPK1 signal profiles plots indicate their lack of association. After stress induction (**lower panels E**–**J**), EPPK1 re-localizes to keratin filaments, demonstrating a stress-dependent association. The almost perfectly overlapping keratin and EPPK1 signal profiles in the corresponding plots indicate their co-localization after stress induction. Scale bar: 10 µm.

To shed light on EPPK1’s localization and function in relation to the keratin intermediate filament cytoskeleton under homeostatic and stress conditions, we generated epithelial cell lines to simultaneously visualize and monitor EPPK1 and keratin by fluorescence microscopy in living cells. To this end, the previously described AK 13-1 cells derived from human vulvar carcinoma-derived epithelial A-431 cells stably expressing human K13-EGFP (HK13-EGFP) [29] were used. The endogenous EPPK1 gene of AK 13-1 cells was fluorescently tagged by insertion of a cDNA fragment encoding mScarlet prior to the stop codon using a CRISPR/Cas9 homology-directed repair strategy (HDR) (Figure 1A and Figure S1). The initial characterization of these cells, named EpiKI/AK, by immuno-blotting showed that they express two full-length EPPK1 fusion protein variants at physiological levels (Figure S2). This is based on the fact that in AK 13-1 cells, two EPPK1 variants can be identified, differing in size due to copy number variations in their almost identical C-terminal PRDs. Comparison of their sizes with the ones of EPPK1 variants in HaCaT cells, which had been determined by PCR before [13], enabled us to estimate their molecular weight as 497 and 672 kDa, respectively (Figure S2). Since higher expression levels of tagged proteins are preferable for long-term live-cell imaging studies, AK 13-1 cells overexpressing human EPPK1 were generated. To avoid recombination events of DNA sequences encoding the almost identical C-terminal PRDs of EPPK1 in *E. coli*, we constructed a cDNA encoding a short variant of EPPK1 consisting only of the first eight N-terminal PRDs and the last of the identical C-terminal PRDs (Figure 1B). Such an EPPK1 variant has been identified recently in the murine genome [14]. We named it “short EPPK1” and the corresponding cell line producing it shEpi/AK. To test for potential differences in the cellular localization of N- and C-terminally-tagged EPPK1 variants, we tagged the short EPPK1 at its N-terminus with mScarlet. ShEpi/AK cells showed a pronounced overexpression of the short EPPK1 variant in immuno-blots (Figure S2). Time-lapse recording of EpiKI/AK and shEpi/AK cells revealed that both N- and C-terminally tagged EPPK1 variants showed completely diffuse localization in the cytoplasm and did not co-localize with keratin filaments (Figure 1C,D, left panels). This finding was unexpected and contradicted the previously reported EPPK1 co-localization with keratin filaments in fixed epithelial cells [16,17,18]. Trying to resolve this obvious discrepancy, we performed live cell imaging of EpiKI/AK and shEpi/AK cells to monitor EPPK1 localization during cell fixation. Adding paraformaldehyde (PFA) at different concentrations induced rapid EPPK1 re-localization to keratin filaments in EpiKI/AK cells (Figure 1C and Figure S3A). Similar results were obtained with shEpi/AK cells (Figure 1D and Figure S3B), though a more intense background of unbound diffuse EPPK1 was detected, which may be explained by the higher expression levels of the short EPPK1. In both cell lines, the extent of co-localization of keratins and EPPK1 was dependent on PFA concentrations being higher at low dosages of the fixative. EPPK1 association with keratin filaments was also detected when methanol fixation protocols were used (Figure S3C). Our findings clearly show that fixation conditions can influence EPPK1 localization in cellular samples leading to wrong conclusions about the actual cellular EPPK1 localization in the native vital cellular environment. The same is true for AK 13-1 cells, as immunofluorescence staining after PFA fixation resulted in a partial keratin filament-association of EPPK1 (Figure S3D).

In previous reports, we and others described EPPK1 as being localized in a keratin-associated pattern in HeLa cells [16], primary hepatocytes [18], and epidermal cell lines [16,17]. However, these reports were inconsistent, describing EPPK1 localization patterns that ranged from fully filamentous [18] to occasionally diffused [16].

### 3.2. EPPK1 Re-Localizes to Keratin Filaments during Stress in a Reversible Manner 

**Figure 2:**
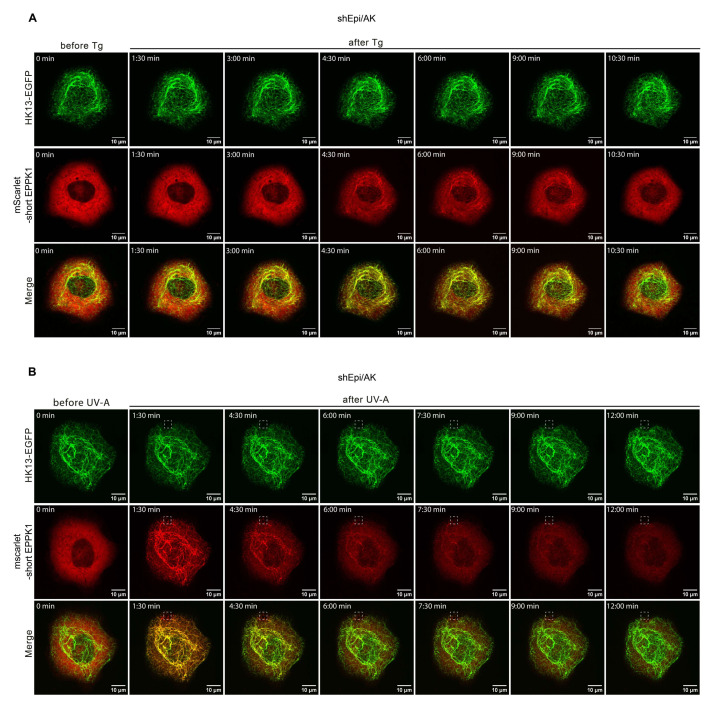
EPPK1 re-localization to keratin filaments after Tg treatment and irradiation with a UV-A laser is reversible. (**A**) Live-cell imaging of shEpi/AK cells treated with 100 nM Tg. Upon treatment, short EPPK1 (red) re-localized to keratin filaments (green) within 4 min and 30 s and returned to a mostly diffuse state within 10 min 30 s after treatment. Images correspond to Video S1. (**B**) Live-cell imaging of shEpi/AK cells partially irradiated with a UV-A pulse of 16 J/cm^2^ in the area indicated by a dashed square. The applied UV-A dosage corresponds to physiological UV-A irradiation levels (up to 60 J/cm^2^) [25]. Images were taken every 90 s, and a z-stack was acquired for every time point. UV-A irradiation was applied once after three images were acquired. Upon UV-A treatment of shEpi/AK cells, short EPPK1 (red) re-localized to keratin filaments (green) within 1 min and 30 s and returned to a mostly diffuse state within 12 min after irradiation. Images correspond to Video S2. Scale bar: 10 µm.

After showing that EPPK1 quickly changes its intracellular localization when cells were treated with fixatives, we wondered whether stress application also influences EPPK1 localization. Live-cell imaging of EpiKI/AK and shEpi/AK cells revealed that EPPK1 re-localization to keratin filaments occurred after thapsigargin (Tg)-induced ER-stress (Figure 1E,H; and Video S1), H_2_O_2_-induced oxidative stress (Figure 1F,I), and partial (Figure 1G,J) or whole cell irradiation (Figure S4) with physiological dosages of UV-A light. These observations were corroborated by analyses of signal intensity patterns in microscopic images (Figure 1E–J). In addition, the stress-induced association of EPPK1 with keratin filaments did not change the overall keratin network morphology. Surprisingly, we could not identify EPPK1 association with keratin filaments after application of osmotic stress by urea (Figure S5), which had been reported for fixed cells before [17]. In contrast to fixation, EPPK1 association with keratin filaments gradually reverted back to a diffuse EPPK1 pattern when sub-lethal dosages were used. An example is shown for shEpi/AK cells that had been treated with Tg (Figure 2A), displaying partial co-localization of EPPK1 with HK13 culminating after 4 min 30 s. In shEpi/AK cells locally irradiated with UV-A light, surprisingly, an almost complete association of EPPK1 with HK13 was detectable throughout the entire cell already after 1 min 30 s (Figure 2B and Video S2). In several experiments, we observed rapid association of EPPK1 and HK13 starting between 30 s and a few min depending on treatment dosages, typically reverting back within 2 to 30 min (data not shown).

Next, we wanted to investigate whether our findings are restricted to AK 13-1 cells or are also valid for other epithelial cell types. We introduced cDNAs encoding shEpi in human MCF7 mammary gland epithelial cells and simple epithelial liver PLC cells overexpressing EYFP-tagged HK14 and EYFP-tagged HK18, respectively [30,31]. In both cell lines (shEpi/MCF7 and shEpi/PK), we could identify the same diffuse EPPK1 localization as in AK 13-1 derived cells, which was quickly changed after the addition of Tg to shEpi/MCF7 and H_2_O_2_ to shEpi/PK cells, respectively (Figure S6A,D), strongly indicating that this feature is common to all epithelial cells expressing EPPK1. In accordance, immunofluorescence microscopy using an EPPK1-specific antibody resulted in a filamentous staining pattern for EPPK1 in MCF7 cells that were fixed with PFA and methanol (Figure S6B,C). Taken together, our data show that EPPK1 translocation to keratin filaments can be elicited by cellular stress, that it is reversible and a common feature of most, if not all, epithelial cell types producing different keratins.

### 3.3. EPPK1 Re-Localization to Keratin Filaments Is Ca^2+^-Dependent

**Figure 3:**
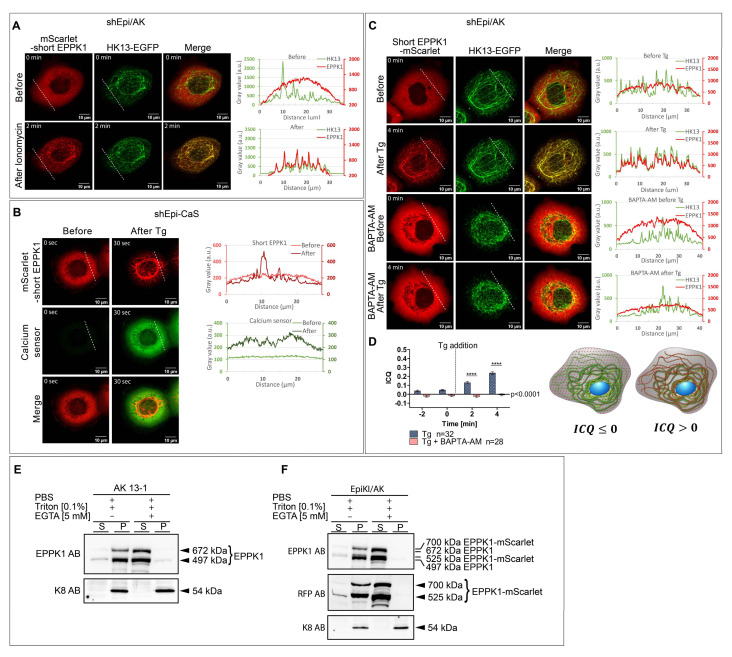
EPPK1/keratin filament association is induced by elevation of intracellular Ca^2+^ levels. (**A**) Live-cell imaging of shEpi/AK cells treated with 165 nM ionomycin. In standard cell culture conditions (**upper panel**), EPPK1 is not associated with keratin filaments but distributed diffusely in the cytoplasm. After ionomycin treatment (**lower panel**), EPPK1 translocated to keratin filaments. Plots on the right side of the panels show fluorescent signal intensities of EPPK1 (red) and HK13 (green) along the dotted white lines indicated in the images. Note the non-overlapping keratin and EPPK1 signal profiles in the absence of ionomycin (**upper panel**) and the almost perfect overlap of both after addition of ionomycin (**lower panel**). (**B**) Single frames from a time-lapse recording of shEpi-CaS cells overexpressing mScarlet-short EPPK1 and the Ca^2+^ sensor GCaMP6m-XC. Under standard culture conditions (**left panel**), EPPK1 (red) shows a diffuse non-filamentous localization pattern and the Ca^2+^ sensor (green) shows only a faint fluorescent signal, indicating low intracellular Ca^2+^ levels. A total of 30 s after the addition of 200 nM Tg (**right panel**), short EPPK1 shows a filamentous localization pattern (red) accompanied by an elevation of Ca^2+^ sensor fluorescence intensity (green), indicating a Ca^2+^-dependent EPPK1 translocation. Plots on the right side of the panels show fluorescent signal intensities of short EPPK1 (red) and the Ca^2+^ sensor (green) along the dotted white lines indicated in the images taken before (light colors) and after Tg treatment (dark colors). The signal profiles for EPPK1 changed from a uniform signal intensity throughout the cell before treatment to a signal profile with several high-intensity peaks after Tg treatment, typical for keratin filament association (compare with Figure 1). In the case of the Ca^2+^ sensor, the signal intensity profile increased after Tg addition, indicating elevated levels of intracellular Ca^2+^. Images correspond to Video S3. (**C**) The Ca^2+^ chelator BAPTA-AM inhibits EPPK1 re-localization. Live-cell imaging of shEpi/AK cells expressing mScarlet-short EPPK1 (red) and HK13-EGFP (green) cultivated in FluoroBrite DMEM medium with and without 20 µM BAPTA-AM (upper two and lower two panels, respectively) before and after treatment with 200 nM Tg. Before Tg treatment (**first panel**), EPPK1 showed a non-keratin-associated diffuse cellular localization, which changed to a filament-associated pattern after Tg addition (**second panel**). Plots on the right side of the panels show fluorescent signal intensities of EPPK1 (red) and HK13 (green) along the dotted white lines indicated in the images. The separated keratin and EPPK1 signal profiles (**upper panel**) almost perfectly overlap after the addition of Tg (**lower panel**) as shown before (see Figure 1H). Cells grown in BAPTA-AM-containing medium showed a comparable diffuse EPPK1 localization pattern before (**third panel**) and after (**fourth panel**) the addition of Tg, indicating that chelation of Ca^2+^ inhibits Tg-induced re-localization of EPPK1. The plots next to the panels displaying the fluorescent signal intensities of short EPPK1 (red) and HK13 (green) corroborate this finding. Note the lower levels of EPPK1 signals when compared with those of HK13 due to stronger bleaching as a consequence of higher laser intensities used for imaging. (**D**) Intensity correlation quotient (ICQ) analysis shows inhibition of HK13/EPPK1 co-localization by BAPTA-AM. A comparison of the ICQ of EPPK1 and HK13 fluorescent signals from control cells and cells pretreated with 20 µM BAPTA-AM before and after Tg treatment is shown. ICQ values were calculated using the intensity correlation analysis plugin of ImageJ (see Section 2). ICQ values vary from 0.5 (for perfect co-localization of signals; see right cell scheme) to −0.5 (for total mutual exclusion of signals; see left cell scheme). Cell schemes show keratin filaments in green and EPPK1 in red. Note that before Tg treatment, ICQ levels of signals from cells in BAPTA-AM containing medium are lower than those of cells cultivated in medium without BAPTA-AM, as all cytoplasmic Ca^2+^ is removed. Data represent at least three independent experiments. Statistical evaluation was performed using the student *t*-test in GraphPad Prism software; ns *p* > 0.05, **** *p* ≤ 0.0001. (**E**,**F**) EPPK1 associates with the insoluble keratin pool in a Ca^2+^-dependent manner. (**E**) Protein lysates from AK 13-1 cells obtained by using the buffers indicated (with and without EGTA) were subjected to a sedimentation assay followed by immunoblot analysis using antibodies against EPPK1 and K8. By comparison with the size of EPPK1 variants determined for HaCaT cells [13] and the known size of mScarlet-short EPPK1 (see Figure S2), we estimated the sizes of the two endogenous EPPK1 variants expressed in tetraploid AK 13-1 cells to 672 kDa for a variant comprising 15 PRDs and 497 kDa for a variant with 12 PRDs, respectively. Supernatant (S) fractions represent soluble, keratin-unbound EPPK1, and pellet (P) fractions represent keratin filament-bound EPPK1. EPPK1 fractionation carried out with a lysis buffer without EGTA induced co-sedimentation with keratins in the pellet (P) fraction. Hardly any EPPK1 signals were observed in the supernatant (S) fraction, indicating that the entire amount of EPPK1 is associated with the insoluble keratin pool during cell lysis. EPPK1 fractionation carried out with a lysis buffer containing the Ca^2+^ chelator EGTA abolished co-sedimentation of EPPK1 with keratin filaments. The entire amount of EPPK1 can be found in the supernatant (S) fraction. (**F**) Protein lysates from EpiKI/AK cells, which were FACS-sorted for mScarlet expression, were prepared by using the buffers indicated (with and without EGTA) and subjected to a sedimentation assay followed by immunoblot analysis using antibodies against EPPK1, RFP, and K8. The sizes of the endogenous EPPK1-mScarlet variants are 700 and 525 kDa, and 672 and 497 kDa for the untagged endogenous EPPK1, respectively. Supernatant (S) fractions represent soluble, keratin-unbound untagged and mScarlet-tagged EPPK1, and pellet (P) fractions represent keratin filament-bound untagged and mScarlet-tagged EPPK1. A similar result as in (**E**) was obtained. Scale bar: 10 µm.

Next, we wanted to identify the trigger for the rapid re-localization of EPPK1 to keratin filaments. As all of the treatments that led to the change of EPPK1 localization increase intracellular Ca^2+^ levels, we wanted to find out whether these two processes are causally linked. To this end, we treated the different cell models with the Ca^2+^ ionophore ionomycin. Indeed, we observed ionomycin-induced re-localization of EPPK1 to keratin filaments in shEpi/AK cells (Figure 3A). Since cytosolic Ca^2+^ seems to be essential for the EPPK1 association with keratin filaments, we reasoned that physiological ligands for ion channels or G-protein-coupled receptors such as ATP or bradykinin should also trigger this effect. In accordance, both ligands were functional in changing EPPK1 localization in shEpi/AK cells (Figure S7A,B), but they only led to a partial and temporary association of EPPK1 with keratin filaments. To further corroborate the involvement of cytoplasmic Ca^2+^, we generated the cell line shEpi-CaS, which overexpresses mScarlet-short EPPK1 together with the Ca^2+^ sensor GCaMP6m. As expected, live-cell imaging revealed that cells under normal growth conditions presented a diffuse EPPK1 pattern and low levels of GCaMP6m fluorescence. However, after Tg treatment, EPPK1 re-localization to keratin filaments coincided with a rapid increase in the Ca^2+^ sensor fluorescence intensity (Figure 3B and Video S3). Concordant observations were made when shEpi-CaS cells were treated with H_2_O_2_ (data not shown), ionomycin (data not shown), ATP (Figure S7C), bradykinin (Figure S7D), and local UV-A irradiation (Figure S8). To confirm that EPPK1 localization is regulated by cytoplasmic Ca^2+^ levels, shEpi/AK cells were grown in the presence of the cell-permeant selective Ca^2+^ chelator BAPTA-AM before Tg treatment. Live-cell imaging showed that 4 min after the addition of Tg, EPPK1 re-localization had taken place, whereas in cells treated with BAPTA-AM, EPPK1 still showed a diffuse cytoplasmic localization pattern (Figure 3C).

An intensity correlation analysis of the fluorescence signals of EPPK1 and keratin corroborated these findings (Figure 3D). Next, we wanted to investigate whether membrane permeabilization-induced Ca^2+^-influx is sufficient to induce EPPK1 re-localization. We performed live-cell imaging of shEpi/AK cells before and after cell lysis with a Triton X-100-containing buffer in the presence or absence of EDTA. Membrane lysis led to EPPK1 re-localization to keratin filaments (Figure S9A and Video S4), although only a part of the EPPK1 molecules associated with keratin filaments while the rest diffused out of the cell. As expected, EPPK1 completely leaked out of the cells in the presence of EDTA (Figure S9B and Video S5). This finding also proves that the entire EPPK1 pool is soluble in the absence of Ca^2+^. To further substantiate a Ca^2+^-dependent interaction of EPPK1 with keratin filaments, we pursued an alternative approach by performing co-sedimentation assays with cell lysates derived from AK 13-1 and EpiKI/AK cells in the presence or absence of Ca^2+^. We could detect WT EPPK1 and EPPK1-mScarlet in the keratin-containing insoluble pellet fractions derived from AK 13-1 and EpiKI/AK cells only when Ca^2+^ was present in the cell lysis buffer (Figure 3E,F). The addition of the Ca^2+^ chelator EGTA to the lysis buffer completely inhibited EPPK1 interaction with insoluble keratins.

### 3.4. EPPK1 Association with Keratin Filaments Slows down Keratin Dynamics

**Figure 4:**
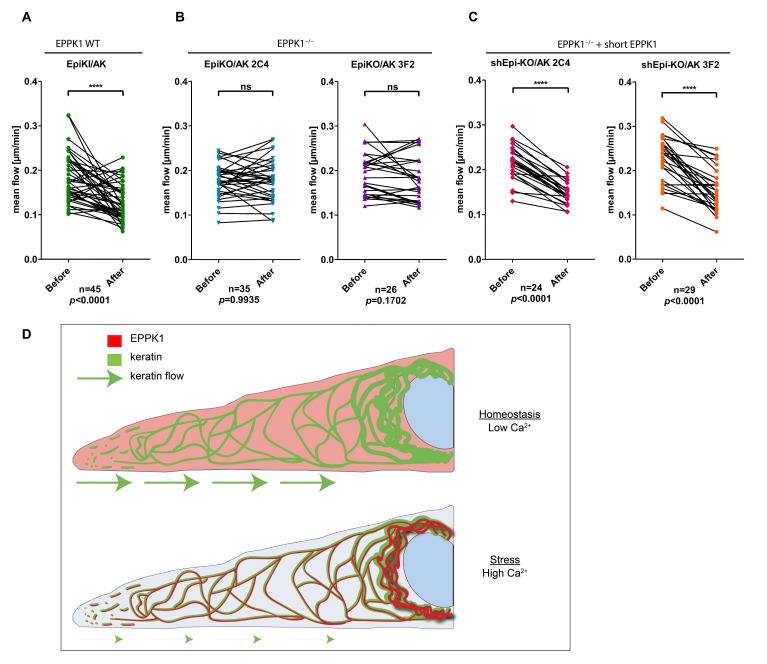
EPPK1-keratin association reduces the mean flow of keratins. Keratin mean flow is significantly reduced in EPPK1 WT cells expressing mScarlet-tagged endogenous EPPK1 (**A**) after Tg-induced binding of EPPK1 to keratins, but it is not slowed down in the two independent EPPK1^−/−^ cell lines, EpiKO/AK 2C4 and EpiKO/AK 3F2 (**B**). The phenotype of EPPK1^−/−^ cells could be rescued in the cell lines shEpi-KO/AK 2C4 and shEpi-KO/AK 3F2 by overexpression of short EPPK1 in an EPPK1^−/−^ background (**C**). Plots show the mean flow of keratin [µm/min] of 10 time points before Tg addition (EPPK1 not associated with keratins) and 10 time points after Tg addition (EPPK1 associated with keratins). CMove was used to calculate keratin flow as described in [26]. Data represent at least three independent experiments. Statistical evaluation was performed using the Wilcoxon matched-pairs signed-rank test in GraphPad Prism software; ns *p* > 0.05, **** *p* ≤ 0.0001. (**D**) Schematic depicting the newly discovered localization and function of EPPK1 in homeostatic and stressed cells.

Next, we wanted to investigate whether the rapid Ca^2+^-induced re-localization of EPPK1 has consequences for keratin filaments. Since it was reported that properties of the keratin cycle are altered during cellular stress responses, we examined whether the association of EPPK1 with keratin filaments influences their dynamics. For this analysis, we generated two independent EPPK1^−/−^ clones of AK 13-1 cells (EpiKO/AK 2C4 and EpiKO/AK 3F2 [see Figure S2]) using CRISPR-Cas9 technology and compared these cells with EpiKI/AK cells, which express tagged EPPK1 at endogenous expression levels. The keratin flow was monitored in single cells before and after the addition of Tg. In EpiKI/AK cells, EPPK1 re-localization to keratin filaments led to a significant decrease in the mean keratin flow (Figure 4A and Video S6). In contrast, when cells of both EPPK1^−/−^ clones were treated with Tg in the same way, no significant influence on keratin dynamics could be detected (Figure 4B and Video S7). We generated cell lines expressing short EPPK1 in the background of both EPPK1^−/−^ cell lines (shEpi-KO/AK 2C4 and shEpi-KO/AK 3F2) to investigate whether this phenotype can be “rescued”. In both cell lines, expression of short EPPK1 led to a significant slowdown of the keratin flow upon Tg treatment (Figure 4C; and Video S8), thereby reverting the non-responding phenotype of their EPPK1^−/−^ mother cell lines.

## 4. Discussion

In this study, we used time-lapse microscopy to investigate the interaction of EPPK1 and keratins in stressed and homeostatic cells. We made the unexpected discovery that EPPK1 proteins are only diffusely distributed in the cytoplasm of different vital cell types under homeostatic, i.e., non-stressed conditions. This observation is in contrast to previous reports for fixed cells, which described a partial or completely keratin filament-associated cellular localization of EPPK1 [16,17,18,21]. Moreover, we find that EPPK1 associates with keratin filaments selectively in situations, which lead to elevated cytoplasmic Ca^2+^ levels. Most importantly, we show that the re-localization of EPPK1 to keratins is extremely rapid and reversible and leads to reduced keratin dynamics.

Specifically, keratin-associated, filamentous localization of EPPK1 has been reported for HeLa, HaCaT, and A-431 cells [16], primary keratinocytes [20,21], and primary hepatocytes [17,18]. However, there was one exception: Jang and colleagues identified a mostly diffuse pattern for EPPK1 in NHEK cells [16]. Interestingly, these authors also reported that not all NHEK cells displayed positive EPPK1 staining. However, when NHEK cells were grown in a high Ca^2+^ medium, EPPK1 colocalized with keratin filaments. We could not corroborate this finding in shEpi/AK cells, which were kept in a similar medium and monitored by live-cell imaging (data not shown). Instead, we found that EPPK1 associates with keratin filaments only when drugs and stresses such as UV-A were applied, which led to elevated intracellular Ca^2+^ levels. This had already been stipulated in a previous report [17], which noted that the cytoplasmic EPPK1 pool was associated with the keratin network of primary keratinocytes after the application of cellular stress, albeit only in situations when EPPK1 was already partially keratin filament-associated. The stresses in this study included, besides UV irradiation, keratin hyperphosphorylation, and osmotic shock. The latter observations, however, are in contrast with our current findings that neither treatment of cells with the phosphatase inhibitor okadaic acid (OA; not shown) nor the hypoosmotic stress inducer urea elicits keratin filament association of soluble EPPK.

What are the reasons for the different findings regarding EPPK1 localization gained by microscopy of fixed versus living cells? Our experiments using different PFA concentrations indicate that the time span between the beginning of fixative-induced membrane permeability for Ca^2+^ and the metabolic inactivation of cellular components determines the degree of EPPK1 association with keratin filaments in homeostatic cells. We propose that these parameters can vary significantly in each experiment, leading to the divergent findings regarding cellular EPPK1 localization reported so far.

Jang and colleagues compared soluble and insoluble pools of EPPK1 during Ca^2+^-induced differentiation of NHEK cells and showed that both EPPK1 pools were increasing but their ratios did not differ dramatically during differentiation [16]. Both soluble and insoluble EPPK1 were also identified in cell fractions from keratinocytes [17], with the soluble pool strongly reduced after treatment with OA. We failed to identify OA-induced EPPK1 re-localization to keratin filaments in living cells (data not shown). How can these obvious discrepancies be explained? We showed that by controlling the amount of Ca^2+^, EPPK1 can almost entirely be shifted from an insoluble to a soluble cellular pool (Figure 3E,F). In addition, lysis in the presence of a Ca^2+^ chelator leads to leakage of all cellular EPPK1 (Figure S9). This may explain why EPPK1 expression was not detected in all cells of a fixed NHEK culture [16]. 

Based on the new findings, we propose that data relating to the amount of keratin filament-associated EPPK1 gained by microscopy or biochemical assays depends on the Ca^2+^ concentration in buffers and media and on the duration of the time span between residual cellular activity and cell death during fixation and lysis. We furthermore conclude that EPPK1 localization data obtained from microscopy of fixed cells or findings regarding the presence or absence of EPPK1 in soluble or insoluble cellular protein pools has to be taken with caution. They are potentially affected by fixation-induced responses of EPPK1 distribution and unintended lysis-induced Ca^2+^ influx, which also alters the amount of keratin filament-associated EPPK1.

Intracellular Ca^2+^ has been shown to play an important role in signaling processes during stress responses and wound healing [32,33]. Interestingly, a direct and quick influence of intracellular Ca^2+^ levels on the actin cytoskeleton organization has been identified for several cell types [34]. Besides drugs used in the experiments presented here, such as ionomycin and thapsigargin, these authors also performed UV laser ablation to damage membranes and induce Ca^2+^ influx-triggered actin reorganization. However, only a few findings have described the impact of intracellular Ca^2+^ on keratin functions so far. An indirect role of Ca^2+^ was indicated by findings which showed that PKC-dependent phosphorylation of K8 [35] and K18 [36] induced keratin network remodeling. So far, we have failed to identify Ca^2+^-induced PTMs of EPPK1 or keratins and cannot rule out a direct impact of Ca^2+^ on the binding properties of both proteins. Studies of envoplakin PRD interactions with vimentin revealed that a broad basic groove recognizes acidic rod elements of vimentin [10,11]. This mechanism may also apply to other members of the plakin protein family [11], and the question arises whether Ca^2+^-dependent changes in their keratin binding affinities are restricted to EPPK1 PRDs or if those found in other plakins show these properties, too. In our opinion, such findings would open entirely new territories for the analysis of the biological roles of these proteins. 

How EPPK1 association with keratin filaments is slowing down their inward movement, which is thought to be driven by interactions with actin and microtubules [4], is an exciting question, as EPPK1 only comprises intermediate filament-interacting PRDs. What is the advantage of a Ca^2+^-induced reduction of the dynamic behavior of keratins by EPPK1 (summarized in Figure 4D)? It could be beneficial for cell survival, for example, by avoiding the uncontrolled formation of keratin aggregates during stress. Such a role could also explain the phenotypes identified in EPPK1^−/−^ mice, which suffered from an aggravated course of disease in the liver and pancreas accompanied by increased formation of keratin aggregates [18,22]. EPPK1-keratin interactions could also aid in Ca^2+^-induced membrane repair after stress-induced damage or strengthen cells mechanically in stress situations.

It has been speculated for a long time that the mammalian genome not only encodes proteins organizing and controlling actin and microtubule filament assembly and organization but also proteins performing comparable functions for intermediate filaments. Based on the data presented in this report, we propose that EPPK1 is one of these long awaited intermediate filament-“controlling” proteins. EPPK1 seems to be unique, as it regulates intermediate filaments only in stressed cells and compromised tissues. The finding that EPPK1 is part of almost all vertebrate genomes after its first appearance 450 million years ago [37] strongly implies that it fulfills important functions that provide an evolutionary advantage. Stress protection would certainly qualify. We are optimistic that our data will help reveal the conditions in which EPPK1 functions are beneficial for organisms and, consequently, will lead to the identification of patients harboring mutations in their EPPK1 genes. Moreover, we expect that the new findings presented in this work will trigger new attempts to study the impact of elevated intracellular Ca^2+^ levels on the functions of intermediate filaments and on the biological role of other intermediate filament-associated proteins of the plakin protein family.

## Figures and Tables

**Table 1:** Cell lines used and generated in this study.

Abbreviation	Origin of Cell Line	Special Features
AK 13-1	A 431	HK13-EGFP
shEpi/AK	AK 13-1	HK13-EGFP mScarlet-short EPPK1
EpiKI/AK	AK 13-1	HK13-EGFP mScarlet-tagged endogenous EPPK1
EpiKO/AK 2C4	AK 13-1	HK13-EGFP EPPK1^−/−^
EpiKO/AK 3F2	AK 13-1	HK13-EGFP EPPK1^−/−^
shEpi-KO/AK 2C4	EpiKO/AK 2C4	HK13-EGFP EPPK1^−/−^ mScarlet-short EPPK1
shEpi-KO/AK 3F2	EpiKO/AK 3F2	HK13-EGFP EPPK1^−/−^ mScarlet-short EPPK1
shEpi-CaS	A-431	mScarlet-short EPPK1-mScarlet Ca^2+^ sensor
shEpi/PK	PK 18-5	HK18-YFP mCherry-short EPPK1
shEpi/MCF7	MCF7	K14-EYFP mScarlet-short EPPK1

**Table 2:** Primary antibody used in this study.

Antibody	Vendor/Source	Reference Number	Target	Species	Dilution
Troma 1	Developmental Studies Hybridoma Bank, University of Iowa	-	Keratin 8	Rat	IB: 1:1000
Anti RFP	chromotek	6g6	mScarlet	Mouse	IB: 1:1000
EPPK1#66	ThermoFisher	PA5-66869	Human EPPK1	rabbit	IB: 1:200
EPPK1#24	Abcam	ab247172	Human EPPK1	rabbit	IF: 1:50

**Table 3:** Secondary antibodies used in this study.

Antibody	Vendor	Species	Dilution
HRPO anti mouse IgGs	Jackson ImmunoResearch Labs	Goat	IB: 1:20,000
HRPO anti rat IgGs	Jackson ImmunoResearch Labs	Goat	IB: 1:20,000
HRPO anti rabbit IgGs	Vector Laboratories	Goat	IB: 1:20,000
Alexa Fluor 647 anti rabbit	Jackson ImmunoResearch Labs	Donkey	IF: 1:500

**Table 4:** Drugs used in this study.

Substance	Vendor	Reference Number	Concentration Used
Thapsigargin	Sigma Aldrich	T9033	200 nM
Hydrogen peroxide	Sigma Aldrich	H1009	2.5 mM
Ionomycin	Sigma Aldrich	I0634	165 nM
ATP	Sigma Aldrich	11140965001	50 µM
Bradykinin-acetate	Sigma Aldrich	B3259	500 nM
BAPTA/AM	Sigma Aldrich	196419	20 µM
Urea	Sigma Aldrich	U5378	300 nM

## Data Availability

Not applicable.

## Supplementary Materials

The following supporting information can be downloaded at: https://www.mdpi.com/article/10.3390/cells11193077/s1, Figure S1 shows a schematic overview of the strategy for the generation of the EpiKI/AK cell line using a CRISPR/Cas9 HDR. Figure S2 shows an immunoblot analysis of EPPK1 expression in the cell lines used. Figure S3 shows EPPK1 re-localization to keratin filaments after fixation with PFA and methanol. Figure S4 shows EPPK1 re-localization to keratin filaments after whole cell irradiation with a physiological UV-A dosage. Figure S5 shows treatment of shEpi/AK cells with urea. Figure S6 shows EPPK1 localization in shEpi/MCF7 and shEpi/PK cells after fixation and application of stress. Figure S7 shows ATP- and bradykinin-induced short-lasting and reversible EPPK1 re-localization to keratin filaments in shEpi/AK and shEpi-CaS cells. Figure S8 shows local irradiation of shEpi-CaS cells with a UV-A laser. Figure S9 shows live-cell lysis of shEpi/AK cells using buffers with and without EDTA. Video S1 shows re-localization of mScarlet-short EPPK1 to keratin filaments after Tg treatment. Video S2 shows reversible mScarlet-short EPPK1 re-localization to keratin filaments after irradiation with UV-A. Video S3 shows live-cell imaging of a shEpi-CaS cell treated with Tg. Video S4 shows live-cell imaging of a shEpi/AK cell before and after cell lysis using a lysis buffer without EDTA. Video S5 shows -live-cell imaging of a shEpi/AK cell before and after cell lysis using a lysis buffer containing EDTA. Video S6 shows EPPK1 association-induced reduction of the mean flow of keratins in EpiKI/AK cells. Video S7 shows that the keratin flow is not slowed down in EPPK^−/−^ cells. Video S8 shows that reintroduction of short EPPK1 into EPPK^−/−^ cells rescues their phenotype by reducing the mean flow of keratin.
