# Supplementary material for: A Ca2+-Mediated Switch of Epiplakin from a Diffuse to Keratin-Bound State Affects Keratin Dynamics
Source: Cells. 2022 Sep 30;11(19):3077. doi: 10.3390/cells11193077 (PMC9563781; doi:10.3390/cells11193077)
Supplement: Supplementary file 1 [file cells-11-03077-s001.zip › cells-1895336-supplementary.pdf]

SUPPLEMENTAL FIGURE LEGENDS

Figure S1. Schematic overview of the strategy for the generation of the EpiKI/AK cell line using a CRISPR/Cas9 HDR. Top: *EPPK1* WT allele with sgRNA target sequence including protospacer-adjacent motif (PAM) marked in red. Middle: *EPPK1* knock-in allele after successful integration of mScarlet (red), and linkers (pink) at the 3'-end of the coding region of the gene. Bottom: Targeting vector used as HDR template. Homology arms (HA) green.

Figure S2. Immunoblot analysis of EPPK1 expression in cell lines. Immunoblots visualizing protein levels of untagged and tagged endogenous EPPK1 and short EPPK1 in AK 13-1 cells and their derivatives. With the exception of AK 13-1 and EPPK$^{-/-}$ cells, all cell lines were FACS-sorted for mScarlet expression before lysis. Proteins were detected using anti-EPPK1 and anti-RFP antibodies. Protein lysates of cell lines expressing untagged or tagged EPPK1 displayed EPPK1 and RFP immuno-positive bands of the expected size. No EPPK1 immuno-positive bands could be detected in EPPK$^{-/-}$ cells, proofing successful elimination of EPPK1. AK 13-1, EpiKI/AK, and shEpi/AK cells are expressing untagged or tagged endogenous EPPK1, cells from the clones EpiKO/AK 2C4, EpiKO/AK 3F2, shEpi-KO/AK 2C4, and shEpi-KO/AK 3F2 are devoid of endogenous EPPK1, and shEpi/AK, shEpi-KO/AK 2C4, and shEpi-KO/AK 3F2 cells express mScarlet-short EPPK1. The number of PRDs and the corresponding sizes of the two endogenous EPPK1 variants expressed in tetraploid AK 13-1 cells (15 PRDs, 672 kDa and 12 PRDs, 497 kDa) were estimated based on comparisons with the EPPK1 immuno-positive bands of HaCaT cells [17 PRDs, 789 kDa and 15 PRDs, 672 kDa (Ishikawa et al., 2018) and with mScarlet-short EPPK1 (9 PRDs+mScarlet, 352.5 kDa)]. The sizes of the endogenous EPPK1-mScarlet variants are 642 and 525 kDa. Note, EPPK1 degradation products are marked with asterisks.

Figure S3. EPPK1 is diffusely distributed in the cytoplasm of living A-431-derived cells and rapidly associates with keratin filaments during fixation with PFA and methanol. (A and B) Live-cell imaging during PFA fixation of EPPK1-mScarlet (red) and HK13-EGFP (green) in EpiKI/AK cells (A) or shEpi/AK cells (B). The respective left panels show cells before fixation, the middle panels cells after removal of medium and subsequent PBS wash, and the right panels cells after 2% and 4% PFA addition. At standard culture conditions, EPPK1 displays a non-keratin filament associated localization. After PFA addition EPPK1 re-locates to keratin filaments. Note that the degree of EPPK1 re-localization to keratin filaments was inversely proportional to the PFA concentration used (compare with Fig. 1 C and D). (C)

Live-cell imaging of shEpi/AK cells fixed with methanol. The upper panel shows mScarlet-short EPPK1 (red) and HK13-EGFP (green) before fixation and the lower panel shows cells after the addition of methanol. EPPK1 re-localizes to keratin filaments after methanol addition. (D) Immunofluorescence microscopy of PFA-fixed, HK13-EGFP-expressing AK 13-1 cells (green), which were stained with an EPPK1 antibody (red). EPPK1-positive signals show a dotty pattern which partially co-localizes with keratin filaments. Scale bar: 10 µm.

Figure S4. shEpi/AK cells show EPPK1 re-localization to keratin filaments after whole cell irradiation with a physiological UV-A dosage. mScarlet-short EPPK1 (red) and HK13-EGFP (green) were monitored in shEpi/AK cells before and after treatment with a physiological dosage of UV-A light using confocal life microscopy and full environmental control. Different representative cells were imaged before and after UV-A irradiation. Scale bar: 10 µm.

Figure S5. Urea treatment of shEpi/AK cells does not lead to association of short EPPK1 with HK13-EGFP. shEpi/AK **c**ells were treated with 300 nM urea during live-cell imaging. Images were taken every 2 minutes, and a z-stack was acquired for every time point. mScarlet-short EPPK1 (red) remains diffused before and after treatment with urea and does not colocalize with HK13-EGFP filaments (green) during the entire duration of the experiment. Note: Urea treatment caused an aggregation of keratins (2 min) which was reversed 6 minutes after urea addition. Scale bar: 10 µm.

Figure S6. Short EPPK1 shows diffuse cellular localization in living mammary gland and simple epithelial liver cells and re-localizes to keratin filaments after stress application and during fixation. (A) Live cell imaging of shEpi/MCF7 cells before and after treatment with 200 nM Tg. Cells overexpress mScarlet-short EPPK1 (red) and HK14-EYFP (green). The upper panel displays short EPPK1 and keratin localizations before treatment, showing that EPPK1 is diffuse and not associated with keratin filaments. The lower panel shows short EPPK1 and keratin association 2 min after treatment. (B and C) MCF7 cells stably expressing HK14 EYFP (green) were stained with EPPK1 antibody (red) after fixation with 4% PFA (B) or methanol (MeOH) (C). In both cases EPPK1 shows filamentous localization which is in clear contrast to the findings with cells under non-stressed conditions (see Fig. S4 A). (D) shEpi/PK cells expressing short EPPK1 tagged with mCherry (red) and HK18 YFP (yellow) showed a diffuse EPPK1 localization. Treatment with 2.5 mM H_2O_2 resulted in the commencement of EPPK1 association with keratin filaments already 15 s after the application of the substance. A complete filamentous EPPK1 pattern was visible 45 s after addition of the drug. Scale bar: 10 µm.

Figure S7. Physiological ligands for ion channels or G-protein-coupled receptors such as ATP or bradykinin trigger short-lasting and reversible EPPK1 re-localization to keratin filaments by elevation of intracellular Ca^{2+} levels. (A and B) Live-cell imaging of shEpi/AK cells before and after treatment with 50 µM ATP (A) or 0.5 µM bradykinin (B). Images were acquired every 30 s. Before the addition of the drugs (A and B most left images), short EPPK1 shows a diffuse localization pattern. At the first monitored time point after drug addition (A and B, second images from the left), short EPPK1 (red) had already re-localized to keratin filaments (green). EPPK1 re-localization was reversible as it returned to a diffuse state a few minutes after treatment. (C and D) Live-cell imaging of shEpi-CaS cells before and after treatment with 50 µM ATP (C) or 0.5 µM bradykinin (D). Images were acquired every 30 s. Short EPPK1 (red) shows a change from a diffuse (C and D, most left images) to a filamentous pattern, which coincided with the increasing fluorescence intensity of the Ca^{2+} sensor (green). EPPK1 started to re-localize back to a diffuse state when fluorescence intensities of the Ca^{2+} sensor went down (C and D most right images). Scale bar: 10 µm.

Figure S8. Local irradiation with a UV-A laser causes a Ca^{2+}-dependent and reversible EPPK1 re-localization to keratin filaments. shEpi-CaS cells were irradiated once with UV-A during live-cell imaging using a UV laser in the area indicated by a dashed square. The applied UV-A corresponds to a UV dosage of 16 J/cm^2 which lies within the field of physiological UV-A irradiation (up to 60J/cm^2) (Gruber et al., 2007). Images were taken every 90 seconds, and a z-stack was acquired for every time point. UV-A irradiation was applied once after three images were acquired. In shEpi-CaS cells treated with the UV-A laser (white square), mScarlet short EPPK1 (red) changed the localization from a diffused to a filamentous pattern which coincided with the Ca^{2+} sensor pGCaMP (green) fluorescence intensity increase, indicating a rise in the intracellular Ca^{2+} concentration in the whole cell. Additionally, EPPK1 re-localized back to a diffuse state when the fluorescence intensity of the Ca^{2+} sensor was decreasing. Scale bar: 10 µm.

Figure S9. shEpi/AK cells show a Ca^{2+}-dependent EPPK1 association with keratin filaments during live cell lysis. (A) Live cell imaging of shEpi/AK cells before and after cell lysis using a lysis buffer without EDTA (50 mM Tris HCl pH 7.4; 0.1% Triton X-100). Cells express mScarlet-short EPPK1 (red) and HK13 EGFP (green). Before the addition of the lysis buffer, EPPK1 showed a non-keratin associated localization in the cell, which changed to a keratin filament-associated pattern after addition of the lysis buffer. Non-keratin associated EPPK1 diffused out of the cell explaining the loss of EPPK1 fluorescence signals after cell lysis.

(B) Live cell imaging of shEpi/AK cells before and after cell lysis using a buffer containing EDTA (50 mM Tris HCl pH 7.4; 0.1% Triton X-100; 5 mM EDTA). Cells express mScarlet-short EPPK1 (red) and HK13 EGFP (green). Before cell lysis, cells showed a non-keratin associated EPPK1 localization. After the addition of lysis buffer, non-keratin associated EPPK1 diffused out of the cell also in this instance. As a consequence, EPPK1 signals could not be detected anymore, showing that the presence of EDTA inhibited Ca^{2+}-induced EPPK1 association with keratin filaments. Scale bar: 10 µm.

VIDEO LEGENDS

Video S1. mScarlet - short EPPK1 re-localizes to keratin filaments after treatment with Tg. A shEpi/AK cell expressing mScarlet-tagged short EPPK1 (red) and EGFP-tagged HK13 (green) was imaged using time-lapse fluorescence microscopy before and after treatment with 200 nM Tg with an interval of 30 s. Time point of drug addition is indicated in the video. The video is a maximum intensity projection of acquired z-stacks. Display rate: 5 frames per second. Scale bar: 10 µm. This video corresponds to images shown in Fig. 2 A.

Video S2. EPPK1 re-localizes reversibly to keratin filaments after irradiation with a UV-A laser. An shEpi/AK cell expressing mScarlet-tagged short EPPK1 (red) and EGFP-tagged HK13 (green) was partially irradiated with a UV-A pulse during live-cell fluorescence imaging using a 355 nm laser (passively Q-switched pulsed ablation laser 16 mW average power, irradiation area 50x50 pixels, 40% laser intensity, 10 ms irradiation time per pixel) in the area indicated by a dashed square. Images were taken every 90 s, and a z-stack was acquired for every time point. UV-A irradiation was applied once after three images were acquired. The start of irradiation is indicated in the video. Upon UV-A treatment of shEpi/AK cells, mScarlet-short EPPK1 (red) re-localized to HK13-EGFP (green) within 1 min and 30 s and returned to a completely diffuse state about 10 min after irradiation. Display rate: 5 frames per second. Scale bar: 10 µm. This video corresponds to images shown in Fig. 2 B.

Video S3. Live-cell imaging of an shEpi-CaS cell treated with Tg. A shEpi-CaS cell overexpressing mScarlet-short EPPK1 (red) and the Ca^{2+} sensor GCaMP6m-XC (green) was imaged using time-lapse fluorescence microscopy before and after treatment with 200 nM Tg. The time point of drug addition is indicated in the video. Under standard conditions, EPPK1

shows a diffuse localization pattern and the Ca^{2+} sensor produces a faint fluorescent signal only, indicating low intracellular Ca^{2+} levels. 30 s after addition of Tg, short EPPK1 showed a filamentous localization pattern accompanied by an elevation of Ca^{2+} sensor fluorescence intensity, indicating a Ca^{2+} dependent EPPK1 translocation. The video is a maximum intensity projection of z-stacks acquired every 15 s. Display rate: 5 frames per second. Scale bar: 10 µm. This video corresponds to images shown in Fig. 3 B.

Video S4. Live-cell imaging of an shEpi/AK cell before and after cell lysis shows re-localization to keratin filaments. An shEpi/AK cell expressing mScarlet-tagged short EPPK1 (red) and EGFP-tagged HK13 (green) was imaged using time-lapse fluorescence microscopy before and after lysis with a buffer containing 50 mM Tris HCl pH 7.4; 0.1% Triton X-100. The time of lysis buffer addition is indicated in the video. Before the addition of the lysis buffer, EPPK1 showed a diffuse localization in the cell, which changed to a keratin filament-associated pattern after addition of the lysis buffer. Non-keratin associated EPPK1 diffused out of the cell, hence the clear loss of EPPK1 fluorescence signals after cell lysis. The video is a maximum intensity projection of acquired z-stacks. Imaging interval: 1 min. Display rate: 5 frames per second. Scale bar: 10 µm. This video corresponds to images shown in Fig. S9 A

Video S5. Live-cell imaging of an shEpi/AK cell before and after cell lysis reveals Ca^{2+}-dependent re-localization to keratin filaments. A shEpi/AK cell expressing mScarlet-tagged short EPPK1 (red) and EGFP-tagged HK13 (green) was imaged using time-lapse fluorescence microscopy before and after lysis with a buffer containing 50 mM TrisHCl pH 7.4, 0.1% Triton X-100 and 5 mM EDTA. Time of lysis buffer addition is indicated in the video. Before cell lysis, cells showed a non-keratin associated EPPK1 localization. After the addition of lysis buffer, no EPPK1 signal could be detected anymore, showing that the presence of EDTA inhibited Ca^{2+}-induced EPPK1 association with keratin filaments. The video is a maximum intensity projection of acquired z-stacks. Imaging interval: 1 min. Display rate: 5 frames per second. Scale bar: 10 µm. This video corresponds to images shown in Fig. S9 B.

Video S6. EPPK1 association reduces the mean flow of keratins. An EpiKI/AK cell expressing mScarlet-tagged EPPK1 (red) and EGFP-tagged HK13 was imaged using time-lapse fluorescence microscopy before and after treatment with 50 nM Tg with an interval of 90 s. Only a representative peripheral part of the cell is shown. Tg was added after 21 min, which is indicated in the video. HK13-EGFP (inverted gray scale) flow is reduced in EpiKI/ AK cells after Tg-induced binding of EPPK1-mScarlet (red) to keratins. The video shows a maximum

intensity projection of acquired z-stacks. Display rate: 5 frames per second. Scale bar: 10 µm. This video shows a representative cell used for the analysis presented in Fig. 4 A.

Video S7. Keratin flow is unaltered in Tg-treated EPPK$^{–/–}$ cells. An EpiKO/AK 3F2 cell expressing HK13-EGFP was imaged using time-lapse fluorescence microscopy before and after treatment with 50 nM Tg with an interval of 90 s. Only a representative peripheral part of the cell is shown. Tg was added after 21 min, which is indicated in the video. HK13-EGFP (inverted gray scale) flow is not reduced in EpiKO/AK 3F2 cells after addition of Tg. The video shows a maximum intensity projection of acquired z-stacks. Display rate: 5 frames per second. Scale bar: 10 µm. This video shows a representative cell used for the analysis presented in Fig. 4 B.

Video S8. Reintroduction of short EPPK1 into EPPK$^{–/–}$ cells rescues the phenotype by reducing the mean flow of keratin. A shEpi-KO/AK 3F2 cell expressing mScarlet-tagged short EPPK1 (red) and EGFP-tagged HK13 was imaged using time-lapse fluorescence microscopy before and after treatment with 50 nM Tg with an interval of 90 s. Only a representative peripheral part of the cell is shown. Tg was added after 21 min, which is indicated in the video. HK13-EGFP (inverted gray scale) flow is reduced in shEpi-KO/AK 3F2 cells after Tg-induced binding of mScarlet-short EPPK1 to keratins. The video shows a maximum intensity projection of acquired z-stacks. Display rate: 5 frames per second. Scale bar: 10 µm. This video shows a representative cell used for the analysis presented in Fig. 4 C.
